# Ramsay Hunt Syndrome: An Introduction, Signs and Symptoms, and Treatment

**DOI:** 10.7759/cureus.33688

**Published:** 2023-01-12

**Authors:** Yuktam Goswami, Sagar S Gaurkar

**Affiliations:** 1 Otolaryngology - Head and Neck Surgery and Surgical Oncology, Jawaharlal Nehru Medical College, Datta Meghe Institute of Medical Sciences, Wardha, IND

**Keywords:** corticosteroids, antivirals, facial paralysis, vesicular rash, varicella zoster virus, geniculate ganglion

## Abstract

Ramsay Hunt syndrome is the complication of the virus varicella-zoster and the infection caused by it, which shows apparent geniculate ganglion involvement. This article discusses the etiology, epidemiology, and pathology of Ramsay Hunt syndrome. Clinically it may be presented as a vesicular rash on the ear or even in the mouth, pain in the ear, and facial paralysis. Some other rare symptoms may also be present, which are also discussed in this article. Skin involvement is also seen in some cases as patterns due to anastomoses between cervical and cranial nerves. This article provides an overview of how the varicella-zoster virus causes facial paralysis and other neurological symptoms. Knowing about this condition and its clinical features is essential to make an early diagnosis and, thus, provide a good prognosis. A good prognosis is required to reduce the nerve damage, prevent further complications, and start an early therapy of acyclovir and corticosteroid. This review also presents a clinical picture of the disease and its complications. The incidence of Ramsay Hunt syndrome has gradually decreased over time because of the development of the varicella-zoster vaccine and better health facilities. The paper also talks about how the diagnosis of Ramsay Hunt syndrome is made and the various treatment options available. Facial paralysis in Ramsay Hunt syndrome presents differently than Bell's Palsy. If not treated for too long, it may cause permanent muscle weakness and may also cause a loss of hearing. It may be confused with simple herpes simplex virus outbreaks or contact dermatitis.

## Introduction and background

The varicella-zoster virus (VZV) persists for life in the spinal and cranial nerve ganglia following primary varicella infection. Any stressful condition can cause reactivation and replication of the virus, which then travels through the sensory nerve fibers and into the dermatome associated with the involved ganglion. Further viral replication in the keratinocytes results in typical clinical features of vesicles in herpetiform distribution [1,2]. Besides the symptoms related to affected dermatomes, the herpes zoster virus can also cause various neurological complications. Segmental paresis can occur when the VZV reactivates in the spinal ganglia; cervical disease may cause upper limb impairment, while the more distal thoracic disease may present as paralytic abdominal wall hernias [2]. In herpes zoster, involvement of cranial nerves may lead to paresis, and ophthalmic and facial nerves are most commonly affected [2,3]. At the beginning of the 20th century, Hunt reported in a series of publications the occurrence of herpetiform lesions on the concha of the ear or oral mucosa in combination with various neurological manifestations [2,4]. James Hunt gathered information from 15 subjects suffering from radiculopathy in which nerve inflammation and edema were seen, including one of his own, in which a resistant and pellucid material deposition in the epineurium is evident [4].

Inflammation of the geniculate ganglion of eight cranial nerves is evident in Ramsay Hunt syndrome. Still, before it was discovered, in patients who presented with ear rash and pain, these symptoms were thought to be associated with inflammation of the Gasserian ganglion or trigeminal nerve (auriculotemporal branch) [4]. Ramsay Hunt syndrome is a late consequence of an infection due to the VZV, which causes inflammation of the geniculate ganglion of the seventh cranial nerve [5]. Less than 1% of varicella-zoster infections result in this syndrome. A characteristic feature of the Ramsay Hunt syndrome is the triad of ipsilateral facial paralysis, otalgia, and vesicular rash in the auricle or auditory canal. However, the first symptom is pain in the affected ear. The vesicles are most commonly seen on the ear but may also be present on the affected area's tongue, cheek, or scalp. There is a variant known as zoster sine herpete, in which the patient presents with severe pain and facial paralysis but no vesicular rash in the affected area and is almost indistinguishable from Bell's Palsy (facial paralysis without inflammation) [6]. Ramsay Hunt syndrome is the second most occurring cause of non-traumatic facial nerve paralysis of the periphery. Before 1986, the incidence of peripheral facial paralysis was estimated, and 4.5% to 8.9% of individuals with peripheral facial paralysis had VZV [6].

Bell's Palsy is also associated with herpes simplex virus, so as a precaution, whether the infection is of herpes simplex virus or VZV, preliminary treatment of antiviral drugs such as acyclovir, famciclovir, valacyclovir for about one and a half weeks should be given along with corticosteroids such as prednisone regardless of whether it is Bell's Palsy or Ramsay Hunt syndrome [4,7,8]. According to recent data for 2022, the incidence of this disease in India is five cases per lakh people every year, whereas the prevalence of Bell's Palsy is 15-30% more than Ramsay Hunt syndrome [9,10]. Factors like stress, chemotherapy, immunocompromised individuals, malnutrition, and infection can increase the severity of the disease and delay recovery [11]. Due to medical advancements, we know that the geniculate ganglion lies close to the vestibulocochlear nerve inside the bony facial canal, which explains some general symptoms like vertigo, nystagmus, tinnitus, nausea, and vomiting. Anyone who has had chickenpox may develop Ramsay Hunt syndrome [12].

## Review

This review article focuses on Ramsay Hunt syndrome, its etiology, signs and symptoms, and treatment. Existing research literature and relevant studies regarding the topic of concern were read and a detailed analysis was undertaken in the indexes of PubMed, Science Direct, Scopus, and Google Scholar. Hardly any language or time constraints were applied. To obtain a detailed search, more articles, synonyms, and derivatives of the phrases were employed; the following evaluation phrases were used: ''varicella zoster virus'', ''facial paralysis'', and ''antiviral therapy''.

Etiology

Ramsay Hunt syndrome is caused by VZV, a member of the human herpes virus family. VZV contains double-stranded deoxyribonucleic acid. Factors that may reactivate the VZV after the primary infection include the person who has never had the chicken pox vaccine, immunocompromised patients, newborns, pregnant women, and any physical or psychological stress [13]. A decrease has been seen in the incidence of chickenpox since the development of the VZV vaccine in 1995; despite that, Ramsay Hunt syndrome has been reported in patients who have never been infected with chickenpox but have been vaccinated with live attenuated VZV [14-16].

Epidemiology

Ramsay Hunt syndrome can occur in anyone who has had chickenpox [13]. It may affect individuals of any age group, but people in the seventh and eighth decades are more susceptible to it, and it is rare in children. Ramsay Hunt syndrome is estimated to account for 16% of all causes of unilateral facial palsies in children and 18% in adults [17]. It is also thought to be the cause of as many as 20% of clinically diagnosed cases of Bell's Palsy [17]. About 7% of cases of Ramsay Hunt syndrome cause acute facial paralysis [18].

Signs and symptoms

**Figure 1:**
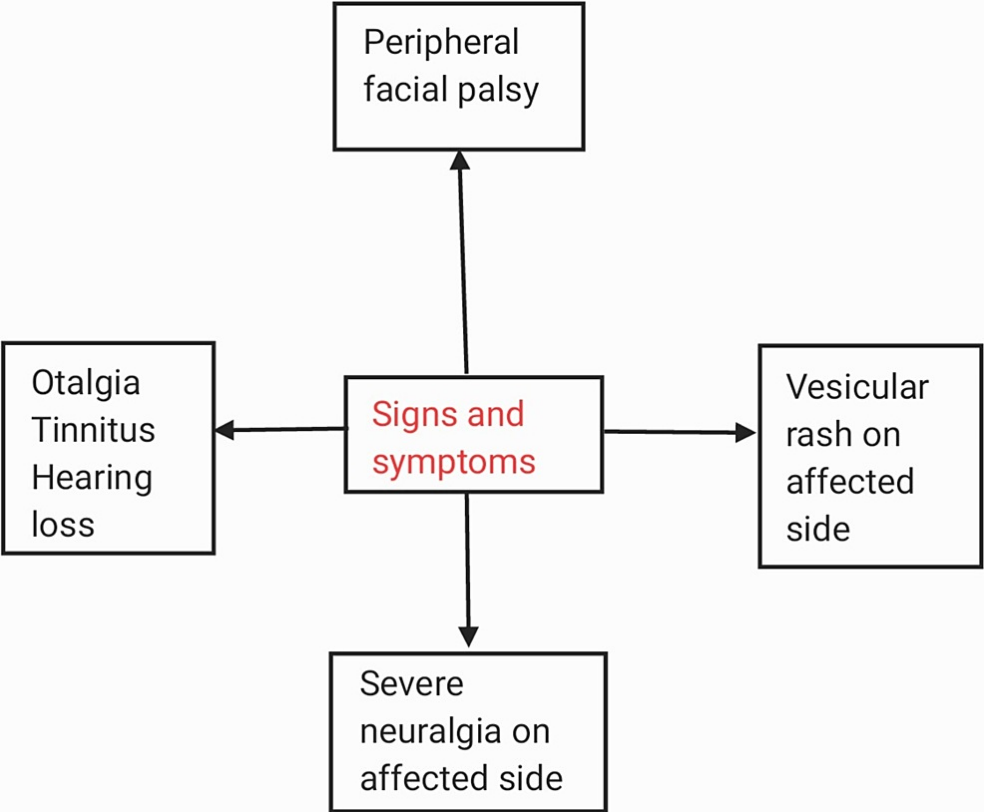
Signs and Symptoms.

Findings in herpes zoster oticus appear in Hunt's Zone (including the tympanic membrane, middle ear, and the cavum conchae). We know of the two primary symptoms of this disease: facial paralysis and rash affecting the ear, but these are not necessary to coincide. In general, the symptoms vary from person to person [4,19,20]. Hunt also contributed to findings such as the severe neuralgias experienced deep in the face scattering to the ear on the affected site, and it also affected the tear and saliva secretion alongside the nasal congestion. These findings were relieved by nervus intermedius sectioning [21]. Changes in taste, dry eyes, hyperacusis, weeping, dysarthria, and nasal obstruction are a few uncommon symptoms. Most of the time, only one side of the face is affected, causing weakness in the facial muscles of that side. It presents as the patient's inability to smile. The severity of this only increases over time, usually a week or two after the onset. This is evident by the dribbling of saliva from the corner of the mouth and the inability to close the eyes later, causing irritation and blurring of vision [12]. The rash is red and vesicular, filled with fluid, and associated with pain affecting the anterior portion of the pinna and the outer one-third of the external auditory canal. The rash can also be seen on some patients' mouths and soft palates. Other symptoms affecting the ear are otalgia, tinnitus, and hearing loss, which are usually temporary but can also be permanent in some rare cases [12] (Figure 1).

The forehead on the affected side is smoother than the non-affected side, which has creases and folds [12]. Since the orbicularis oris muscle is also paralyzed, the person cannot close the eyes, increasing the distance between the upper and lower eyelid. The lip axis is tilted towards the non-affected side of the face. Facial asymmetry is visible, difficulty in speaking develops, and the flow of secretions such as saliva and tears are reduced. Ramsay Hunt syndrome is known to be a non-contagious infection. But the virus can reactivate, causing chickenpox in people who have not been vaccinated against it or who have not yet had it; thus, people are advised to avoid contact with such patients until the rash scabs off [12]. Isolated lesions on the external ear in 40.6% of patients, as well as combined involvement of the outer ear and external ear and external auditory canal in 25.3%, were found by Walther [22]. Both dermatological and neurological findings lead to the diagnosis of Ramsay Hunt syndrome. The facial nerve is predominantly a motor nerve innervating facial muscles with only a few sensory nerve fibers. Apart from facial paralysis, facial nerve palsy may also present with paralysis of the stapedius muscle of the middle ear, causing hyperacusis [20,23]. If the early presenting clinical features worsen, the probability of cranial nerve damage being permanent increases [24].

Histopathology

The fluid substance is obtained from vesicles under the microscope, revealing giant cells with multiple nuclei and eosinophilic bodies when stained with Giemsa stain, Wright's stain, or methylene blue. This technique is known as the Tzanck smear technique, and it is used to differentiate between types of herpes viruses and conditions such as Pemphigus Vulgaris, leprosy, etc. [25]. The clinical history and neurological examination are understood to be the grounds of the Ramsay Hunt syndrome diagnosis. Microscopic examination of the cerebrospinal fluid (CSF) and magnetic resonance imaging (MRI) enhanced with gadolinium have not been of any diagnostic or prognostic use [26]. The virus is not cultured from ganglia, but by methods such as in situ hybridization, Southern blot analysis, or polymerase chain reaction (PCR), VZV may be detected [27]. Many authors have indicated by the variable histopathological findings that Hunt's theory of geniculate ganglions is not true; instead, it is the viral attack of the facial nerve itself. It is also better identified clinically as laboratory testing is comparatively slower. For more successful management, the doctor must start antiviral-corticosteroid therapy early before the spread of the infection.

Evaluation

Routine testing is not usually recommended as the diagnosis of Ramsay Hunt syndrome is typically made based on clinical history and physical examination [11]. Ramsay Hunt syndrome's typical features include rash, pain, and facial drooping. A Tzanck smear can be performed on fluid obtained from the vesicles, and PCR analysis of tears, saliva, or fluid from the vesicles is available in some academic settings [28]. The degree of cranial nerve involvement can be determined by investigations like audiometry, vestibular testing, and flexible fiberoptic laryngoscopy [28].

Treatment and prevention

It heals in a self-automated way; hence the aims of the treatment are for us to reduce the occurrence of complications such as facial paralysis and post-herpetic neuralgia, to provide immediate relief from the pain and inflammation, and to prevent exposure to keratopathy of the cornea of the affected side as the patient is unable to close the eye [28]. In such cases, ocular ointments have been valuable in keeping the eye lubricated, artificial tears, and wearing an eye patch. Clinically it has been seen over the years that preliminary treatment and good prognosis prevent post-herpetic neuralgia because the elderly population is more susceptible to being immunocompromised; hence an aggressive approach is advised for the treatment [28]. Capsaicin is United States Food and Drug Administration (FDA) approved for the neuropathic pain associated with post-herpetic neuralgia [12]. Various studies have found that oral antivirals and steroids effectively reduce late complications [7]. Now whether the drugs lessen the severity or longevity is still not known. Antivirals such as acyclovir, valacyclovir, and famciclovir have been found useful [28].

Acyclovir 500 mg five times a day is the most affordable option [28]. Valacyclovir is given 1000 mg three times a day and is most effective in Bell's Palsy. Famciclovir, when given 500 mg three times a day, is known to be more effective than acyclovir and is the most affordable option. The treatment is continued for up to a week or 10 days. Still, because it has been reported in some cases that delayed degeneration of the facial nerve axon took up to three weeks, antiviral therapy is preferred to be continued till the given time. The potency of antiviral treatment is disputable when it comes to the paresis of the cranial nerve [4,29-31]. Corticosteroids are administered in high doses, at the same time, for three weeks. Oral prednisone 60 mg is given daily for three weeks and is stopped gradually and not suddenly to avoid acute adrenal complications (Table 1). Corticosteroids are known to have various side effects about which the doctor is expected to guide his patient throughout the treatment. Side effects include irritability, gastric reflux, insomnia, hyperglycemia, etc. Corticosteroids and their value can be illustrated by relief in edema and by decompression of the neurogenic structures that are present in the facial nerve canal in the petrosal bone [4,32,33]. Kinishi conducted an antiviral-corticosteroid combined therapy among 91 patients, of which three-fourths showed promising signs of nerve sensitivity recovery [29]. When he ran only corticosteroid treatment in 47 patients, 53% showed encouraging signs of recovery, significantly less than the antiviral-corticosteroid combined therapy [29].

**Table 1:** Various Studies Conducted and Their Outcomes.

Authors	Year	Type of Study	Outcome	Comments
Uscategui et al. [31]	2008	Randomized control study	Combined antiviral-corticosteroid therapy was found to be more fruitful than corticosteroids alone.	Shortening of viral shedding duration and relief in the symptoms were better with antivirals combined with corticosteroid therapy.
Kinishi et al. [29]	2001	Cross-sectional study	Better nerve function in patients treated with antiviral-corticosteroids combined rather than corticosteroids alone.	Antiviral-corticosteroid combined therapy when done the recovery rate was 75% in contrast to 53% of corticosteroid therapy.
Murakami et al. [9]	2004	Retrospective study	Patients who received the treatment early (within three days) had a better chance (75%) of complete recovery than those who received the treatment 4-7 days later (48%) and (30%).	The recovery rate and the earliness of receiving the treatment were directly proportional to each other.

The study of Uscategui stands against the studies of other authors [31]. Note that fragile diabetics who take hypoglycemic medications must alter their ongoing diabetic course to tolerate the high-dose corticosteroids. Treatment, when started even when delayed by seven days, has still been successful in patients who failed to consult their doctors early. Despite randomized controlled prospective trials conducted, it was found that prednisone and acyclovir are together found to alleviate the prognosis overall. Microvascular decompression and rhizotomy are therapeutic alternatives that have been put aside for cases of resistant neuralgia [4,34-37].

Further treatment is aimed at providing symptomatic relief from pain and rashes; carbamazepine is given to prevent seizures, anticholinergics and antihistamines are given to suppress vertigo, and diazepam also helps relieve pain and dizziness. Please note that these medications are issued only when a patient encounters specific symptoms shown above [11]. Patients with risk factors like hypertension, diabetes mellitus, old age, and some other immunocompromised states need to be careful as they make take more time to get better and should be advised to keep their eyelid weighted on the affected side to ease the closure of the eye and prevent exposure keratopathy during treatment [38]. Corneal hypesthesia is an indication of weight placement on the eyelid of the affected side [39]. Surgical procedures are not helpful in patients with Ramsay Hunt syndrome. Prevention from the VZV can be achieved by administering a vaccine against chickenpox in childhood, or if someone is 50 years or older, they are advised to get the shingles vaccine. These vaccines are remarkably effective against the VZV, which would, in turn, prevent the infection causing the Ramsay Hunt syndrome [12].

Prognosis

Most patients recover from Ramsay Hunt syndrome, but the degree to which their recovery occurs varies from patient to patient [28]. For patients who do not recover, their premorbid function may lead to complications like synkinesis [28]. In general, the prognosis for Ramsay Hunt syndrome is worse than that for Bell's Palsy, as it has a lower rate of synkinesis development [40]. The severity of facial paralysis decides the prognosis of Ramsay Hunt syndrome. However, age over 50, greater axonal damage, presence of oropharyngeal lesions, multiple cranial neuropathies, and diabetes are some other factors that may influence the prognosis of a patient [38]. Besides synkinesis, a common complication of Ramsay Hunt syndrome is post-herpetic neuralgia, in which pain persists for longer than three months after the onset and is more likely to develop in patients older than 50 years [41].

Discussion

Hunt explained three syndromes, out of which the second, the Ramsay Hunt syndrome, is an overdue complication of the VZV infection [28]. It usually affects the old and immunosuppressed individuals with factors that enhance their susceptibilities, such as skeletal status, stress, and comorbidities like diabetes and hypertension [28]. The condition is treatable, but the treatment should not be delayed as it may cause irreversible weakness in the facial muscles and hideous scars due to vesicular rashes on the face [38]. The causative agent is the VZV. The preferred and effective treatment for this condition is combined antiviral and corticosteroid therapy which starts showing improvement within a week [28].

Antivirals used are acyclovir, valacyclovir, and famciclovir, and corticosteroids used are prednisone (orally), etc. If the condition is delayed, severe complications occur, such as corneal damage due to exposure to keratopathy, permanent facial paralysis on one periphery, and scars. It may also cause an inability to have eye closure on the affected side. However, chickenpox vaccine administration in children and shingles vaccine administration in the population aged 50 years or more has been proven quite significant. In medicine, few clinical conditions present with both facial paralysis and facial rashes. Still, Ramsay Hunt can be confused with other conditions, such as local face rashes caused by the herpes simplex virus or impetigo caused by staphylococci. Contact dermatitis caused by poison ivy or neomycin is often confused with Ramsay Hunt since it causes rashes [11,42].

Another differential diagnosis is Bell's Palsy, as it is the second most common cause of acute facial paralysis, similar to zoster sine herpete. Other differentials include Lyme, strokes, ear diseases, malignancy in extratemporal regions, autoimmune diseases, etc. [11,39]. Strokes that spare the forehead are cortical strokes, but stroke in the brainstem region affects the half side of the face. However, presenting symptoms of a stroke include unstable vital signs and neurological symptoms. Various cranial neuropathies may also show up in the central nervous system and not only in infections caused by a virus, which include severe acute respiratory syndrome coronavirus 2 (SARS-CoV-2) [43,44]. With so many advancements and treatable options to choose from while treating a patient of Ramsay Hunt syndrome, we now know that the person would eventually recover and the condition is treatable, but in patients who have comorbidities and are immunocompromised, especially in their old age, can be susceptible to further complications [45,46]. Cutaneous and visceral infections range from light rashes like chickenpox to severe and fatal illnesses. The condition may affect the cranial nerves in proximity to the affected ganglion, which is geniculate. It may spread even further to tangle the central nervous system. Spinal cord inflammation may cause paresis in the bowel and bladder mechanism and the extremities, and in sporadic cases, encephalitis is also seen. Ocular complications lead to irreversible damage to the cornea as the patient cannot close the eye, and exposure keratopathy occurs [47]. The person may also go into depression and have decreased social cues because the vesicular rashes may cause scarring. With facial disorder, the person develops anxiety and remains socially underconfident.

## Conclusions

Ramsay Hunt syndrome is supposed to be a clinical entity affecting the facial nerve and causing paralysis and a rash, paralysis affects only the periphery rather than the whole face, and the rash is also localized to the diseased side. It is caused by the reactivation of the VZV, and in other words, those who have had chickenpox in their lives are susceptible to suffering from Ramsay Hunt syndrome. However, it only affects five people in a lakh, so not everyone who has had chickenpox develops Ramsay Hunt later. People in old age and with multiple comorbidities are the most affected population and are treated with antivirals and corticosteroids combined for the best and the fastest results. People show signs of recovery within three to seven days; in most cases, people suffering recover 100%. Still, this syndrome has quite a few complications ranging from a mild rash to severe palsy. The trick is to seek treatment as early as possible upon encountering the doubtful symptoms and not let the complications occur. Antivirals used are acyclovir, famciclovir, valacyclovir, etc., and corticosteroids such as prednisolone are used.
